# Genetic and environmental influences on adolescent attachment

**DOI:** 10.1111/jcpp.12171

**Published:** 2013-11-21

**Authors:** Pasco Fearon, Yael Shmueli-Goetz, Essi Viding, Peter Fonagy, Robert Plomin

**Affiliations:** 1 Clinical, Educational & Health Psychology, Division of Psychology & Language Sciences, University College London, London, UK; 2 King’s College London, MRC Social, Genetic & Developmental Psychiatry Centre, Institute of Psychiatry, London, UK

**Keywords:** Adolescence, attachment, genetics, environmental influences

## Abstract

**Background:**

Twin studies consistently point to limited genetic influence on attachment security in the infancy period, but no study has examined whether this remains the case in later development. This study presents the findings from a twin study examining the relative importance of genetic and environmental influences on attachment in adolescence.

**Methods:**

The sample included 551 twin pairs aged 15 years recruited from the larger Twins Early Development Study (TEDS). Attachment was assessed using a semistructured interview, the Child Attachment Interview.

**Results:**

We found robust associations between MZ twins’ scores for Coherence and their overall security of attachment (*r* = .42, *p* < .001; kappa = .26, *p* < .001), but substantially lower associations for DZ twins (*r* = .20, *p* = .001; kappa = .09, *p* = .20), suggesting genetic influence on adolescent attachment (and substantial nonshared environment). Model-fitting analyses confirmed this impression, indicating approximately 40% heritability of attachment and negligible influence of the shared environment.

**Conclusions:**

The results suggest that genes may play an important role in adolescent attachment and point to the potentially distinct aetiological mechanisms involved in individual differences in attachment beyond early childhood.

## Introduction

The security of the parent–child attachment relationship has been found to be of considerable significance for children’s emotional development and is thought to exert a continuing influence on socioemotional adjustment across childhood, adolescence and adulthood (Kobak, Cassidy, Lyons-Ruth, & Zir, 2006). Longitudinal studies have demonstrated, for example, that secure attachment, measured in infancy, childhood or adolescence, is associated with better social competence and a lower risk of emotional or behavioural disturbance (Fearon, Bakermans-Kranenburg, van Ijzendoorn, Lapsley, & Roisman, 2010; Lyons-Ruth, Alpern, & Repacholi, 1993). Understanding the causal influences on attachment across the life span is thus an important goal for developmental science and for the advancement of intervention and prevention programmes.

Strong theoretical arguments have been advanced regarding the causal antecedents of attachment security and insecurity and these have focussed heavily on the role of the environment. Indeed, attachment researchers have largely assumed that the quality of parenting, particularly the degree to which the parent is sensitive and responsive to the child’s attachment cues, is the pre-eminent causal factor in the development of individual differences in attachment. Quantitative behavioural genetics provides the most powerful methodology currently available for examining genetic and environmental influences on complex human traits, and has demonstrated the relative ubiquity of genetic influence on childhood personality, emotionality and psychopathology (Plomin, DeFries, Knopik, & Neiderhiser, 2013).

Only fairly recently have researchers used behavioural genetic methods to test the strong hypothesis advanced by attachment theorists regarding the primary role of the environment in individual differences in attachment security. Notably, these studies have quite consistently found evidence of environmental influence on attachment, precisely as predicted by attachment theory. For example, Bokhorst and colleagues (Bokhorst et al., 2003) measured child–parent attachment at 12 months in a sample of 157 twin pairs, using the Strange Situation Procedure and found that 52% of the variance in security was attributable to shared environment, and 48% to nonshared environment, leaving the estimate of heritability effectively at zero (Bokhorst et al., 2003). These findings were not only notable because of the apparent absence of genetic effects, in stark contrast to other domains of development studied previously; they were also surprising because they indicated quite strong shared environmental influence, something not commonly seen in behavioural genetic studies, but predicted by attachment theory. Furthermore, the shared environmental variance in attachment security overlapped substantially with shared environmental variance in observed maternal sensitivity in a manner highly consistent with attachment theory (Fearon et al., 2006).

Two other twin studies, employing different measures of attachment in preschoolers, have yielded estimates of little genetic influence and strong shared environmental influence that are quite consistent with these findings (O’Connor & Croft, 2001; Roisman & Fraley, 2008). Several candidate gene studies have reported associations or gene-by-environment interactions in relation to attachment security in infancy, primarily involving polymorphisms in the dopamine D4 receptor gene, the serotonin transporter gene and the oxytocin receptor gene (Barry, Kochanska & Philibert, 2008; Chen, Barth, Johnson, Gotlib, & Johnson, 2011; Lakatos et al., 2000; Spangler, Johann, Ronai, & Zimmermann, 2009). However, none of these candidate gene associations has been consistently replicated and the most recent paper on the subject, combining two relatively large cohorts, found no evidence of reliable single gene associations or gene-by-environment interactions (Luijk et al., 2011). The twin findings, combined with the lack of reliable findings from association studies, have thus collectively provided important empirical support for a fundamental tenet of attachment theory.

However, as longitudinal studies have found evidence of quite limited continuity in attachment from infancy to later childhood, adolescence or adulthood (Pinquart & Silbereisen, 2002), it is not possible to extrapolate the behavioural genetic data from infancy to later points in development. In addition, when behavioural genetic studies have found evidence of shared environmental effects on development in other domains, they have tended to involve younger samples (infants and preschoolers), suggesting that shared environmental effects may be relatively restricted to infancy/toddlerhood and, when present, may not always be stable beyond the early years (Plomin et al., 2013). Furthermore, it is possible that genes could come to influence attachment in later development because the child’s genes increasingly influence parental attitudes and caregiving behaviour (gene-environment correlation). Indeed, it has been argued that genes may play more of a role in later attachment status because security is measured and conceptualised very differently in adolescence and adulthood. At later ages, the operationalisation of attachment organisation is centred on the way in which individuals think about their attachment relationships rather than on individual differences in observed attachment behaviours. The ability to think coherently about, and reflect upon, early attachment experiences, is the hallmark of secure attachment status beyond early childhood. Main (1996) argued that this ability to reflect upon and integrate what might be difficult early experiences may draw on personal attributes that are partly heritable (Main, 1996). For both conceptual and empirical reasons, there is thus a great need to test the role of genes and environment in individual differences in attachment security beyond the early years.

Adolescence represents a key period in the life span for attachment, in part because it may represent a phase in which Internal Working Models of attachment become consolidated and converge on their adult pattern of organisation (Allen & Land, 1999). There is also good evidence that attachment security when measured in adolescence is linked with overall adjustment and risk for psychopathology (Allen, Porter, McFarland, McElhaney, & Marsh, 2007; Scott, Briskman, Woolgar, Humayun, & O’Connor, 2011). Despite the absence of behavioural genetic studies of attachment as traditionally measured by developmentalists at this age, several studies have examined the role of genes and environments in individual differences in young adults’ self-reported attachment *styles*. This operationalisation of attachment is different from that typically used by developmental researchers, and focuses on conscious feelings of anxiety about a romantic partner’s availability (attachment anxiety) and tendencies to avoid and feel uncomfortable with closeness in adult romantic relationships (attachment avoidance, see Hazan & Shaver, 1987). In a sample of 239 adult twin pairs, Brussoni, Lang, Livesey and Macbeth (2000) estimated that 37% of the variance in attachment security (i.e. lack of attachment anxiety) was due to genes and 60% to nonshared environment. Interestingly, attachment avoidance, by contrast, showed no genetic influence, and 29% of the variance was attributable to shared environment. These results were independently replicated by Crawford et al. (2007), finding 40% heritability for attachment anxiety and none for avoidance. Picardi, Fagnani, Nisticò, and Stazi (2011) using a relatively large sample of young adult twins (*n* = 677 twin pairs), replicated the findings for attachment anxiety (45% heritability; 55% nonshared environment), but not those for avoidance (finding 36% heritability, and 64% nonshared environment). However, findings using questionnaire measures of attachment, while interesting in their own right, cannot be used to unequivocally infer the pattern of heritability of attachment in adolescence or adulthood as measured using representational/interview measures because numerous studies show the former and the latter to be essentially orthogonal (Roisman et al., 2007).

Two studies have examined attachment in nontwin siblings using representational measures, which yield evidence pertinent to questions regarding family resemblance in attachment in adolescence and young adulthood. Both found distinctly low rates of sibling similarity in attachment security. Fortuna et al. (2011) used a continuous scoring of the Adult Attachment Interview (AAI) with 60 young adult siblings and found no significant sibling correlation for the Dismissing dimension (representing the most prevalent feature of insecure adult attachment, see Bakermans-Kranenburg & van IJzendoorn, 2009), although the correlation for Preoccupation was significant (*r* = .33). No significant sibling correspondence was found for overall security when coded categorically with the most commonly used coding scheme by Main and Goldwyn (George, Kaplan, & Main 1996). Similarly, Kiang and Furman (2007) found no significant sibling correspondence for attachment security as measured using the AAI in 41 adolescent siblings. While sibling studies do not allow an estimation of genetic influence, these two studies do suggest that the pattern of environmental and genetic influence on adult attachment may be quite different to that observed in infants and preschoolers. In particular, they suggest that shared environmental influences on adolescent and adult attachment are likely to be modest, although the relatively small samples involved in these two studies preclude strong conclusions being drawn.

In this study, we report findings from a relatively large twin study (*N* = 551 twin pairs) designed to investigate the behavioural genetics of attachment in adolescence, using a well validated representational measure, the Child Attachment Interview (CAI). The study is the first to examine the behavioural genetics of attachment outside the first 3 years of life using a rigorous interview-based procedure designed to assess state of mind with respect to attachment in adolescence. In so doing, this study addresses a crucial issue regarding the determinants of attachment security and insecurity during a period of development of great importance for understanding the emergence of psychopathology.

## Method

### Participants

*The Twins Early Development Study* (TEDS) is a large longitudinal cohort of twins studied intensively since infancy. From a pool of 16,810 twin pairs born between 1994 and 1996, 12,000 returned initial information, and sample sizes in recent cohorts have varied between 6,900 and 5,900 twin pairs. The sample has remained reasonably representative of the UK population (see Trouton, Spinath, & Plomin, 2002). Twin zygosity was diagnosed on the basis of physical similarity and questionable cases were verified with analysis of DNA markers (Kovas et al., 2007).

#### Current study sample

Participants were 582 same-sex twin pairs with an average age of 15. Only same-sex twins were used in the present analyses to avoid potential inflation of genetic estimates when opposite-sex DZ twins are included with same-sex DZ twins. All families participating in TEDS who lived within the greater London area or in urban centres with good transport links to the London area were initially approached to take part in the study. Of the 1292 families who were of the appropriate age (15 years ± 14 months), 694 initially agreed to participate representing 54% of those approached. Of those, 582 were subsequently assessed. The study sample included 320 female twin pairs and 262 male twin pairs. Mean age at assessment was 15 years (range 13.9–16.4 years). Twenty-eight cases had missing information regarding twin zygosity, and 3 further cases were missing CAI interviews due to technical problems. The final sample with known zygosity and complete CAI interviews was 551 twin pairs.

The majority of the families were white (83%) with a median household income of £30,000–£50,000. Over half of the families had both parents in full or part time employment (63%), 31% had completed secondary school and 34% were educated to degree level. The study sample was more educated, more likely to be employed and had a higher household income than the national average obtained from the Office of National Statistics. Based on data obtained at first assessment in the TEDS Study, the families that agreed to participate were more educated than those that were invited but did not take part (31% of the study sample had only high school qualifications relative to 42% of those that were invited but did not take part, overall association χ^2^(7) = 63.9, *p* < .001). Those that took part were not different to those that did not in terms of white versus nonwhite ethnicity (χ^2^(1) = .46, *p* = .50).

### Measures

#### The Child Attachment Interview

The CAI is a semistructured interview which takes between 30 and 60 minutes to complete, designed to assess attachment organisation in middle childhood and adolescence (Shmueli-Goetz, Target, Fonagy, & Datta, 2008). It is informed by the well-established and extensively validated Adult Attachment Interview (AAI, George et al., 1996) with some notable, developmentally sensitive, differences. The CAI comprises 19 questions included to elicit representations of current attachment relationships with primary caregivers. Children’s perceptions of, and experiences with, their attachment figures are sought, with questions focusing on times when children are more likely to call upon their attachment figures such as times of emotional upset, illness, separation and loss. The emphasis is on assessing children’s ability to construct a coherent narrative regarding their current attachment relationships. The interviews are videotaped and transcribed verbatim and the coding process is based on a careful analysis of both verbal and nonverbal communication. The CAI coding and classification system borrows from the AAI (George et al., 1996) although it focuses on recent memories and appraisals rather than more distant early experiences. CAI narratives are rated on several 9-point scales (e.g. Emotional Openness, Anger, Idealisation), including overall Coherence, which is the primary indicator of secure attachment. The system also yields four classifications: Secure, Dismissing, Preoccupied and Disorganised, which are assigned according to expected patterns on the above rating scales as well as an overall evaluation of how well the narrative fits a prototypical profile, as defined in the coding manual. The CAI has excellent test-retest reliability over a 3-month and 1-year period (Shmueli-Goetz et al., 2008). Both CAI classifications and overall coherence have shown good test-retest reliability and criterion validity, correlating robustly with indices of psychological adjustment and differentiating community from clinic-referred children with effect sizes in the range *d* = .60–.70 (Scott et al., 2011). The CAI shows good discriminant validity, as security and coherence are not correlated with verbal IQ, expressive language skills, SES, age, or ethnicity (Shmueli-Goetz et al., 2008). Furthermore, in two independent studies, the CAI was strongly predicted by maternal security of attachment using the AAI (Jacobson and Yumoto, 2009; Shmueli-Goetz et al., 2008).

For this study, the TEDS research assistants were trained by one of the authors (YSG) in the interviewing and coding of CAIs. All coders achieved 80% or higher agreement for attachment classifications from a standard reliability set. For an additional 59 interviews from the current sample, inter-rater reliability was calculated with YSG serving as the gold standard. Twenty-seven of these were complex cases identified for consensus coding with the second author who coded them blind prior to discussion. A further 32 cases were chosen at random for reliability purposes. The inter-rater reliability (intraclass correlation) for coherence was .72. Inter-rater agreement for classifications was 85% and 86% for the secure–insecure split with respect to mother and father respectively (kappa = .69 and .72). Reliability for 3-way classifications was 80% and 83% for mother and father (kappa = .66 and .72) and 75% and 78% for 4-way classifications (kappa = .62 and .67). In a very large percentage of twins (94%), the same classification was assigned for mother and father, and hence in this report we used the maternal classifications.

Of 1116 interviews, 579 were classified as Secure, 429 Dismissing, and only 60 and 32 Preoccupied and Disorganised respectively. Because the number of twins classified as Preoccupied and Disorganised was small, the twin analyses based on the classifications were conducted using the standard secure versus insecure (Dismissing, Preoccupied, & Disorganised) categorisation.

### Procedure

Family history and contact details were obtained from the TEDS database and initial contact was by phone. All participants provided informed consent. The CAIs were conducted at one of two testing sites or in the family home. Of the 582 assessed, 430 were seen at one of the two centres and 152 at home. It was not possible to ensure that interviewers were entirely blind to zygosity. However, each interviewer was entirely blind to the *content* of the interview of the cotwin, as the interviews were conducted at the same time by different researchers. Coding of the interviews was completed independently by two coders who were not the interviewers, were entirely blind to zygosity and had no knowledge of the content or coding of the cotwin’s interview. The study was approved by the University of Reading Research Ethics Committee.

### Data analysis

After presenting basic descriptive data, the primary analyses focus on standard quantitative genetic modelling of attachment, as indexed by the continuous Coherence scale and the overall secure versus insecure classifications. Standard univariate twin modelling uses structural equation modelling techniques to estimate the proportion of variance in a trait that is attributable to additive genes (latent variable labelled A), Common Environment (latent variable labelled C) and NonShared Environment (latent variable labelled E). Structural equation modelling of twin data using raw data maximum likelihood model-fitting allows for tests of relative fit of alternative models, and provides estimates of genetic and environmental parameters and standard errors using all of the data simultaneously (Neale & Cardon, 1992). Modelling begins by testing the fit of the most general genetic and environmental model which includes all three parameters (A, C and E) and then proceeds by testing the reduction in model fit when the genetic and common environmental terms are dropped from the model (the term E is always retained). The difference in −2*Log Likelihood (−2LL) between the saturated model and a nested submodel is distributed as chi-squared which can be used to test hypotheses. The best fitting model is typically taken to be the one with the fewest number of parameters that can be achieved without significantly reducing model fit, as well as the model that minimises Akaike’s Information Criterion (AIC). For the attachment classifications, we estimated (using Maximum Likelihood with robust standard errors) genetic and environmental parameters using the liability threshold model, which assumes that observed categories represent underlying continuous liabilities upon which thresholds have been imposed (Neale & Cardon, 1992). In this case, the difference in −2LL between nested models does not follow a strict chi-squared distribution, so Satorra’s scaled chi-squared difference test was used (Satorra & Bentler, 2010). Following Prescott’s (Prescott, 2004) approach, all models were estimated while covarying for twin gender and age. All models were fit using MPlus 7.0 (Muthén & Muthén 1998–2012).

## Results

The results are presented in two sections. In the first, we present descriptive statistics on the means, variance and proportions of the key indicators of attachment security derived from the CAI, and present measures of the twin–cotwin associations for MZ and DZ twins that form the basis of formal genetic modelling.

### Descriptive statistics

Descriptive data regarding the Coherence scale are provided in Table 1. The twin intraclass correlations were 0.42 (*p* < .001) for MZ twins and 0.20 (*p* = .001) for DZ twins, suggesting genetic influence and no influence of shared environment. For MZ twins, the association between twins’ attachment security was highly significant (kappa = .26, χ^2^(1) = 19.86, *p* < .0001). In contrast, for DZ twins the corresponding association was nonsignificant (kappa = .09; χ^2^(1) = 1.99, *p* = .16) (Table S1 reports correspondence for 3-way and 4-way classifications for completeness). Standard twin probandwise concordances for twin pairs in which at least one member of the twin pair was classified as ‘insecure’ were 44% for MZ twins and 34% for DZ twins (see Table 2). The difference in the MZ and DZ concordances also suggest genetic influence. The contribution of genetics to the twin concordance plus the high base rate of ‘insecure’ in this sample (47%) suggests no role for shared environmental influence. Structural equation model-fitting results for these data are described in the following section.

### ACE twin modelling

The associations described above, though indicative of genetic effects, do not provide direct estimates of genetic and environmental effects. We thus proceeded to conduct formal tests of the role of genes and environment in adolescent attachment using structural equation models. We began with the continuous Coherence scale because continuous scales maximise power to detect genetic and shared environmental effects. The results of the ACE modelling are shown in Table 3. As the table shows, the best fitting model was the AE model, according to the AIC criterion, similar to the results gleaned from the MZ and DZ twin correlations in Table 1. Furthermore, deletion of the shared environment parameter (C) from the ACE model led to a nonsignificant reduction in model fit (Δχ^2^(1) < .01, *p* = .98), while deletion of the genetic parameter from the ACE model led to a significant decrease in model fit (Δχ^2^(1) = 7.70, *p* = .005). The AE model yielded estimates of genetic influences on Coherence of 38% (95% Confidence Interval [CI] = 31%–46%). Nonshared environmental effects were estimated at 62% (95% CI = 54%–69%).

The AE model was also the best fitting liability threshold model for the two-way categorical attachment data (secure vs. insecure) according to the AIC criterion, again similar to the results gleaned from the MZ and DZ twin concordances from Table 2. When the common environment term was removed from the ACE model, no significant decline in model fit was observed (Δχ^2^(1) = .001, *p* = .99), but removal of the genetic term led to a significant reduction in model fit (Δχ^2^(1) = 5.11 *p* = .02). Under the AE model, genetic effects were estimated to account for 35% of the variance in security (95% CI = 22%–48%), and 65% of the variance was estimated to be due to the nonshared environment (95% CI = 52%–78%). For security with fathers, the results were virtually identical (unsurprisingly, given the 94% correspondence between security with mother and father), with heritability estimated at 37% (95% CI = 24%–50%) and nonshared environment 63% (95% CI = 50%–76%).

## Discussion

The results of this study were strikingly different to those that have previously been obtained using twin methodology in samples of infants and toddlers (Bokhorst et al., 2003; O’Connor & Croft, 2001; Roisman & Fraley, 2008). These earlier studies had indicated with considerable consistency that attachment in early life is strongly, if not exclusively, influenced by the environment. Furthermore, they pointed to the important role of the shared environment, in a manner that was highly consistent with predictions from attachment theory. In this study, using a relatively large sample of adolescent twins, strong evidence of genetic influence on attachment was found, and estimates of shared environment were effectively at zero. The estimates of heritability we obtained for the scale representing narrative Coherence and the overall 2-way attachment classification (secure vs. insecure) were around 38% and 35% respectively, with the remaining variance being attributable to nonshared environment and measurement error. It is important to note that these heritability estimates are likely to be conservative, as unreliability of measurement would tend to lead to an underestimation of genetic effects and an overestimation of the nonshared environment.

These findings are highly noteworthy because they are based on a relatively large, well-powered sample – meaning the estimates of heritability are quite precise – and because the tools used represent what many in the field of attachment would consider the most valid way to measure attachment in adolescence (Shmueli-Goetz et al., 2008). Furthermore, very rigorous blind coding was employed, effectively ruling out the possibility that the coding could have created artefactual patterns of association between MZ and DZ twins. While caution must always be exercised when generalising twin findings to other populations, it is also noteworthy that the degree of correspondence we observed in our DZ twins was consistent with two other studies that have used the AAI with adult and adolescent siblings, which lends further weight to the findings.

Our findings call for a reconsideration of the assumptions generally held by attachment theorists regarding the causal influences that shape attachment in adolescence. Our findings indicate that the child’s inherited characteristics have a substantial influence on attachment—as indicated by the way they represent and think about attachment relationships—at this age. While the view that attachment security is driven by the quality of parental care is highly consistent with observational studies and behavioural genetic evidence in early development, the picture is clearly more complex in adolescence. At this stage, we can only speculate about the mechanisms that might be at play, but we tentatively suggest that genetic factors in the child may progressively bias the organisation of attachment between infancy and adolescence. In light of the emerging evidence of substantial change in attachment across childhood, and the limited continuity from infancy to adolescence and adulthood, it is tempting to suppose that genetic factors might become particularly influential during phases of developmental reorganisation and change. One major reorganisation that may be of particular significance is the transformation that presumably occurs when attachment shifts from a primarily behavioural and relational construct (where children may display different attachment patterns with different caregivers for example, see Steele, Steele, & Fonagy, 1996), to one that is more cognitive in nature and more like a generalised style or ‘state of mind’. There is still some uncertainty about when this transformation occurs, but data using the CAI (Shmueli-Goetz et al., 2008) with younger samples suggest that it may be well underway in middle childhood if not before.

Another possibility is that later in development children’s genetic propensities begin to systematically evoke changes in the relative insensitivity of care provided by their primary attachment figures, which in turn leads to changes in the children’s feelings of security in the parental relationship. These, possibly bidirectional, mechanisms would lead one to expect to observe influences of children’s genes on parental care and that these would be associated with, and predate, security of attachment in adolescence. There is good evidence that several dimensions of parenting in adolescence and in earlier development show influence from the child’s genes (e.g., O’Connor, Deater Deckard, Fulker, Rutter, & Plomin, 1998; Pike, McGuire, Hetherington, Reiss, & Plomin, 1996). However, it remains to be seen whether these evoked parenting mechanisms can partly account for genetic variance in attachment security. Finally, we note that if a similar pattern of genetic and environmental influence on attachment also emerged in adulthood, this would raise new and important questions regarding the mechanisms leading to intergenerational concordance in patterns of adult attachment (e.g., Benoit & Parker, 1994), and the possibility that genetic mechanisms may in part explain the concordance of attachment patterns between parents and their adult offspring. This hypothesis should be investigated in longitudinal, genetically informative studies.

This study, like the twin studies of attachment conducted in earlier development, also highlighted the significance of the nonshared environment. Even if one takes account of measurement unreliability, approximately half of the variance in attachment security may be attributable to unique environmental experiences that make twins different, not similar, to each other. Comparatively little work has been done to elucidate the nonshared environmental mechanisms involved in attachment security and insecurity (though see Roisman & Fraley, 2008) and this remains an important area for future research. It would be valuable in future studies to examine experiences that are unique to, or experienced differently by, a particular child within a family, such as parental differential treatment or sibling–sibling conflict, in order to understanding how nonshared variance in attachment in adolescence arises.

This study had several limitations that need to be considered when interpreting the results. First, the sample we studied was relatively middle-class and under-represented more disadvantage communities, which limits the generalisability of the findings. Second, like other adolescent samples, we observed very low rates of Preoccupied and Disorganised/Unresolved attachment and hence the heritability estimates reported here apply primarily to the contrast between secure attachment and dismissing attachment, this being the most common type of insecure attachment in adulthood and adolesence. To date, longitudinal attachment studies indicate that infants classified as disorganised tend to present as dismissing in adolescence, as assessed by the AAI (see for example, Weinfeld, Whaley & Sroufe, 2004). It is not clear whether this reflects a developmental shift to an organised strategy or a methodological artefact. Few adolescents in our sample had experienced abuse or significant loss, which no doubt partly accounts for the low rates of Unresolved attachment. The low proportion of preoccupied attachment in this sample is consistent with studies with adolescents and young adults using the AAI (e.g., Weinfield, Sroufe, & Egeland, 2000). Further behavioural genetic work on attachment in adulthood will be important to clarify the role of genes and environments in patterns of continuity and change in attachment between adolescence and adulthood.

In summary, this study found strong evidence for the role of genetic factors in the development of attachment in adolescence. The degree of heritability was quite substantial and stands in stark contrast to findings obtained in infancy. The findings suggest that as attachment changes during the course of development, genes may play an increasingly important role. The challenge for future research in this area is to elucidate how both genetic and environmental factors within families progressively canalise development towards adaptive or maladaptive patterns of attachment over time.

## Supplementary Material

Table S1

## Figures and Tables

**Table 1:** Descriptive statistics and covariance matrix for CAI Coherence for MZ and DZ twins

	MZ		DZ	
	Twin 1	Twin 2	Twin 1	Twin 2
Descriptive statistics				
Mean	5.15	5.17	5.22	5.20
*SD*	1.74	1.77	1.79	1.64
*N*	288	288	261	261
Covariance matrix				
Twin 1	3.02	0.42^b^	3.22	0.20^b^
Twin 2	1.28^a^	3.12	0.59^a^	2.70

a Covariance.

b Correlation.

**Table 2:** Cross-tabulation by zygosity for CAI secure versus insecure attachment status

	Twin 2	
Twin 1	Secure	Insecure
MZ		
Secure	97	53
Insecure	53	85
DZ		
Secure	80	52
Insecure	67	62

**Table 3:** ACE Model-Fitting Statistics for Coherence and Binary Attachment Security

	Model Statistics				Model parameter estimates		
Models	−2LL	*df*	*p*	AIC	A	C	E
Coherence							
ACE Model	4,237.2	12	.84	4,249.1	.38	.001	.62
AE Model	4,237.2	13	.89	4,247.1	.38	–	.62
CE Model	4,244.8	13	.32	4,254.7	–	.29	.71
Security (Binary Classification)^a^							
ACE Model	2,237.0	9	–	2,249.0	.35	0	.65
AE Model	2,237.01	10	–	2,247.0	.35	–	.65
CE Model	2,241.2	10	–	2,251.3	–	.23	.77

a For categorical outcomes, chi-squared model fit statistics are not available. For nested model comparisons, see text.

## Key Points

- Attachment security is important for life span social and emotional functioning, and is thought to be caused by environmental factors
- Twin studies in infants and toddlers have found evidence of strong environmental influence on attachment, but no studies have been conducted in later development
- Attachment security in adolescence shows quite substantial genetic influence
- The findings suggest that the child’s heritable traits influence attachment, and may indicate that attachment security emerges through the two-way interplay between the child’s genes and the caregiving environment
- The results suggest that efforts to promote adolescent attachment security may need to focus on these two-way processes, and consider how heritable traits influence how adolescents perceive, feel about and respond to, family interactions and relationships
