# Supplementary material for: Genetic and environmental influences on adolescent attachment
Source: J Child Psychol Psychiatry. Author manuscript; Available in PMC 2015 Mar 20. (PMC4366883; doi:10.1111/jcpp.12171)
Supplement: Table S1 [file NIHMS62675-supplement-Table_S1.docx]

Supplementary Table 1 Twin cross-tabulations for MZ and DZ twin pairs for 4-way and 3-way CAI attachment classifications

|  |  | Twin 2 | | | |
| --- | --- | --- | --- | --- | --- |
|  | Twin 1 | Secure | Dism | Preoc | Disorg |
|  | *4-way classification* | | | | |
| MZ | Secure | 97 | 40 | 9 | 4 |
|  | Dism | 47 | 66 | 6 | 0 |
|  | Preoc | 3 | 4 | 2 | 0 |
|  | Disorg | 3 | 3 | 2 | 2 |
|  |  | | | | |
| DZ | Secure | 80 | 40 | 8 | 4 |
|  | Dism | 54 | 42 | 7 | 2 |
|  | Preoc | 9 | 6 | 0 | 1 |
|  | Disorg | 4 | 4 | 0 | 0 |
|  | *3-way classification* | | | | |
| MZ | Secure | 98 | 43 | 10 | -- |
|  | Dism | 49 | 70 | 9 | -- |
|  | Preoc | 3 | 4 | 2 | -- |
|  |  |  |  |  |  |
| DZ | Secure | 80 | 44 | 9 | -- |
|  | Dism | 56 | 46 | 8 | -- |
|  | Preoc | 11 | 7 | 0 | -- |

*Note:* For MZ twins the association between twins’ attachment classifications was highly significant (4-way kappa = .26, *χ*^2^(9) = 48.92, *p* <.0001; 3-way kappa = .25, *χ*^2^(4) = 24.66, p <.0001). In contrast, for DZ twins the corresponding associations were non-significant (4-way: kappa = .06; *χ*^2^(9) = 5.87, *p* = .75; 3-way: kappa = .06; *χ*^2^(4) = 3.53, *p* = .47).
